# Atlantic City in 1901

**Published:** 1900-09

**Authors:** 


### ATLANTIC CITY IN 1901.

FLUSHED with the victory of one of the greatest triumphs in Association history—the amalgamation of three State societies in one strong body—New Jersey feels that there is yet one thing needful for the completion of the glory of this union, and that is a visit of the American Veterinary Medical Association in 1901. They offer as a convention city the most wonderful seaside Mecca in the world—Atlantic City. They tender, through the generous spirit of Captain John Young, an ocean pier for the meeting-place, with every facility for convention work, and every attraction of an ocean resort that has charmed and entertained every profession and calling, every strata of society that has passed through the hospitable door of a city whose latch-string is ever out. New Jersey has never had a meeting, and her claims and influence will be potential in the battle for place in 1901. The President of her State Association is known among the profession as a tireless worker, and a stronger Association advocate is not known among his colleagues; and he will go in to win, backed by his organization, who will only know defeat when the vote is cast and their defeat consummated. Other convention cities for 1901 will need to be up and doing and work early and late.

